# Revitalizing your sleep: the impact of daytime physical activity and balneotherapy during a spa stay

**DOI:** 10.3389/fpubh.2024.1339689

**Published:** 2024-07-08

**Authors:** Lucia Castelli, Andrea Michele Ciorciari, Letizia Galasso, Antonino Mulè, Francesca Fornasini, Angela Montaruli, Eliana Roveda, Fabio Esposito

**Affiliations:** ^1^Department of Biomedical Sciences for Health, University of Milan, Milan, Italy; ^2^Free University of Bozen-Bolzano, Faculty of Education, Bolzano, Italy; ^3^GB Hotels, Abano Terme, Italy

**Keywords:** mud application, thermal treatments, thermal water, active lifestyle, body temperature, exercise

## Abstract

**Background:**

In modern society, achieving high-quality sleep is increasingly challenging. We conducted a study to explore the potential benefits of daytime physical activity and balneotherapy, including mud application and thermal-water bathing, on sleep quality.

**Methods:**

To assess daytime physical activity and sleep parameters, we actigraphically monitored 127 healthy participants (34.6% male, average age 64.61 ± 0.89 years) during a one-week stay at a spa resort, where they received mud application and thermal-water bathings.

**Results:**

Participants were divided into three groups based on the timing of mud application. Those receiving mud application before 8:30 a.m. tended to have shorter sleep durations compared to those with later application, especially if it occurred before 7:45 a.m. However, mud application did not significantly affect sleep quality. Three-way ANCOVA revealed a significant effect of daytime physical activity on delta Sleep Efficiency, but *post-hoc* tests were insignificant. Furthermore, analyzing the duration of daily thermal-water bathings, individuals bathing for over 75 min per day experienced a noteworthy improvement in sleep quality, particularly in terms of delta Sleep Efficiency (2.15 ± 0.9% vs. −0.34 ± 0.31%, *p* = 0.007).

**Conclusion:**

Our findings suggest that extended thermal-water bathing may enhance objective aspects of sleep quality. Since balneotherapy is mainly prescribed for individuals with musculoskeletal pathologies or psychological disorders, these findings may encourage doctors to recommend bathing in thermal water also to healthy subjects. Future researchers need to investigate the role of daytime physical activity in depth.

## 1 Introduction

Proper sleep quality is vital for human health, but modern society faces increasing sleep problems and their negative consequences (1). A recent study reports that sleep problems range between 22.1 and 10.5% in Europe, depending on the country (2), whereas in Italy, insufficient sleep is raised by 30% in the general population (3). Sleep quality can deteriorate due to various reasons, both physiological and pathological. For example, aging (4), stressful and anxious states (5), pathologies (6, 7) and environmental or work conditions (8, 9) could damage sleep quality. From the opposite point of view, long-lasting reduced sleep quality may also predispose individuals to or exacerbate chronic and degenerative diseases or pathologies (10). Therefore, maintaining appropriate sleep quality could be considered a helpful possibility for preserving general physical and mental health (10). In this context, effective and efficient actions and solutions to improve sleep health and quality are welcomed and needed (11, 12).

Physical activity and an active lifestyle are frequently recommended non-pharmacological approaches to enhance sleep quality (13–15). Physical activity encompasses both structured and non-structured physical activities throughout the day, i.e., all daytime activities across various areas of life, such as work, home, and leisure (daytime physical activity) (16, 17), affecting sleep differently. While the impact of structured physical activity on sleep is well-documented across different ages (14, 18–20) and both in healthy and pathological conditions (21–23), research on daytime physical activity’s influence on sleep is relatively new and has yielded mixed results. Some studies suggest a positive association between higher daytime physical activity and improved sleep quality (24–29), while others have found no such connection (30, 31). Discrepancies may arise from the use of different assessment tools, such as questionnaires with subjective measures versus objective measures like accelerometers with different evaluated parameters (activity counts, number of steps, mean acceleration, etc.) (24, 25, 27–31).

Balneotherapy, which involves immersion in mineral or thermal waters and other treatments like mud application and inhalation, has gained interest for its potential to improve sleep quality (32–37). It is commonly used as a complement treatment for musculoskeletal degenerative pathologies and psychological disorders (38–42). Despite the interest in these approaches, there is a lack of objective sleep assessments, particularly in combination with daytime physical activity, in Italian resorts, as well as limited research on healthy individuals (35). Most studies have focused on individuals with health issues, where sleep quality improvements following balneotherapy could be attributed to symptom relief rather than the therapy’s direct effect on sleep (35).

To address these gaps, the current study aims to evaluate the impact of daytime physical activity and balneotherapy, including mud application and bathing in thermal-water pools, on sleep quality in healthy individuals during a 1-week stay in northwest Italian spa resorts.

## 2 Materials and methods

### 2.1 Study design

Recruitment for this study took place between November 2021 and December 2022. The spa center’s medical hydrologist assessed the health status of the eligible participants. Upon acceptance for study participation, participants provided informed consent and completed sociodemographic questionnaires covering gender, age, height, weight, occupation, marital status, and smoking habits. Participants were also equipped with actigraphs to record their daytime physical activity and sleep during their week-long spa visit, which included prescribed mud treatments and access to thermal pools (Supplementary material 1). All procedures were performed in accordance with the 1964 Helsinki Declaration and its later amendments. The study was approved by the University of Milan’s Ethical Committee (24/20).

### 2.2 Participants

The study involved Italian spa resort customers who voluntarily booked a week of balneotherapy stay and treatments. The enrolment included healthy individuals aged 20–80, regardless of gender, who spent at least 1 week at one of the five *GB-Hotel* group spa resorts. After the medical check with the spa’s medical hydrologist, eligible participants were invited to participate in the study if they met the following inclusion criteria: absence of cardiovascular, neuromuscular, endocrine and metabolic pathologies, mobility issues, pregnancy or breastfeeding, use of medication affecting sleep, and diagnosed sleep disorders.

### 2.3 Actigraphic monitoring

The actigraph *Motion Watch 8 (Cambridge Neurotechnology, Cambridge, United Kingdom)* recorded daytime physical activity and sleep data over the entire week. The *MotionWare* software (version 1.2.28) evaluated sleep and daytime physical activity. The validity and reliability of this device and software have been widely reported in the literature, including studies with older populations (43–45).

Participants were instructed to wear the device on their non-dominant wrist during the day, removing it during mud treatments and thermal-water pool baths. A daily diary was used to document actigraph removal, bedtime, and waking time for a more precise analysis of daytime physical activity and sleep data. Participants also recorded the time and duration of thermal-water pool bathing.

Daytime physical activity data were collected as a percentage of actigraph usage time, excluding nighttime hours (waking window). The waking window was determined by checking bed and wake-up times from the dairy. For the statistical analysis, we calculated the tertiles of daytime physical activity to categorize the participants into *low*, *medium* and *high* levels.

Sleep parameters were assessed based on the parameters listed in Supplementary material 2.1 (46), with the first night excluded from analysis due to potential environmental factors. Mean values for each sleep parameter were calculated for the entire week, and delta values (Δ) were computed to evaluate changes between the *first period* (mean of the second, third, and fourth nights) and *the second period* (mean of the fifth, sixth, and seventh nights) of the spa stay.

### 2.4 Balneotherapy

Balneotherapy was carried out at one of the five *GB-Hotel* group spa resorts in Abano Terme, Italy, with details about mud and thermal-water features provided in Supplementary materials 2.2, 2.3. The medical doctor prescribed 15-min mud applications, available between 6:00 a.m. and 12:00 p.m., requiring reservations to capture each participant’s specific application time. For the statistical analysis, we calculated the tertiles of mud application to categorize the participants into *very early*, *early*, and *late* applications.

Bathing in thermal-water pools was optional, but the medical hydrologist recommended bathing three to four times daily for 30 min. Usage details were documented in a diary. For the statistical analysis, we calculated the tertiles of the daily duration of thermal-water bathing to categorize the participants into *short*, *intermediate*, and *long* durations.

### 2.5 Statistical analysis

IBM SPSS Statistics (version 28) was used for statistical analyses. A significance level of α = 0.05 and a 95% confidence interval were employed.

Data were presented with means, standard errors (SE), and numerical or percentage values for continuous and categorical variables, respectively. Body Mass Index (BMI) calculation and categorization were outlined following WHO guidelines (47).

Tertiles were calculated as follows:

- daytime physical activity:

- *low*: ≥19.2% - < 39.5%;
- *medium*: ≥39.5% - < 42.4%;
- *high*: ≥42.4% - ≤ 73%;

- mud application:

- *very early*: ≥06:25 a.m. - < 07:45 a.m.;
- *early*: ≥07:45 a.m. - < 08:30 a.m.;
- *late*: ≥08:30 a.m. - ≤ 11:20 a.m.;

- daily duration of thermal-water bathing:

- *short*: ≥20.3 - < 55.4 min;
- *intermediate*: ≥55.4 - < 75 min;
- *long*: ≥75 - ≤ 167.5 min.

All analyses were adjusted for sex, BMI, and age. Initially, a three-way ANCOVA assessed the main effects of daytime physical activity, mud application time, and thermal-water bathing duration on weekly mean sleep parameters, with subsequent adjustments for mud application time.

A two-way ANCOVA evaluated the main effects of daytime physical activity and thermal-water bathing duration, along with their interactions, on Δ sleep parameter values. Bonferroni *post-hoc* tests were applied when significant effects were found. Effect size (*d*; Cohen’s interpretation) (48) and partial eta-squared (*η_p_^2^*) were calculated.

## 3 Results

### 3.1 Sample size calculation

We used GPower software (version 3.1.9.7) for the sample size calculation. Considering the three-way ANCOVA analysis with 3 covariates (sex, BMI, and age), an effect size of 0.04 with a power of 0.95, an α error of 0.05, and the 95% confidence interval, the software suggested a sample size of 121 participants. To account for potential dropouts and incomplete data for some participants, we initially recruited 151 subjects and ultimately analyzed 127 of them, surpassing the sample sizes suggested by the software.

### 3.2 Descriptive data

Out of the initially recruited 151 individuals, the current study included 127 participants (64.61 ± 0.89 years) (Supplementary material 1). Notably, half of the participants (51.1%) were within the normal weight range, although average BMI slightly exceeded the overweight threshold (25.21 ± 0.39 kg/m^2^). The majority of participants were female (65.4%); considering this disproportion, we opted to adjust all the analyses by sex. All the descriptive data for the study sample can be found in Supplementary material 3 (Supplementary Tables 1, 2).

### 3.3 Effect of mud application time on the weekly mean sleep parameters

The effect of mud application time was the only significant effect revealed by the three-way ANCOVA, which disclosed significant effect of mud application time for *Sleep end* [*F*_(2, 125)_ = 24.66, *p* < 0.001, *ƞ_p_*^2^ = 0.4], *Time in Bed* [*F*_(2, 125)_ = 14.37, *p* < 0.001, *ƞ_p_*^2^ = 0.28], *Assumed sleep* [*F*_(2, 125)_ = 12.94, *p* < 0.001, *ƞ_p_*^2^ = 0.26], *Actual Sleep time* [*F*_(2, 125)_ = 8.54, *p* < 0.001, *ƞ_p_*^2^ = 0.19], and *Immobile minutes* [*F*_(2, 125)_ = 10.76, *p* < 0.001, *ƞ_p_*^2^ = 0.22].

In detail, the Bonferroni *post-hoc* tests revealed that participants who had the mud application *very early* in the morning woke up earlier (*p* < 0.001*, d =* −3.29; *p* < 0.001*, d =* −2.53, respectively) spent less time in bed (*p* = 0.017*, d =* −1.81; *p* < 0.001*, d =* −4.1, respectively), slept less (*p* = 0.038*, d =* −1.76; *p* < 0.001*, d =* −2.89, respectively), and had fewer immobile minutes (*p* < 0.001*, d =* −1.44; *p* = 0.014*, d =* −0.6, respectively) compared to those who had the mud application *early* and *late* in the morning. Additionally, participants with an *early* mud application woke up earlier than those with a *late* mud application (*p* = 0.014*, d =* −0.8) (Table 1). The effects of daytime physical activity, duration of thermal-water bathing, and their interactions were not found to be significant.

**Table 1:** Mean and standard error of the weekly mean sleep parameters stratified for the tertiles of the mud application time.

Sleep parameters	Mud application time		
	Very early (Mean ± s.e.)	Early (Mean ± s.e.)	Late (Mean ± s.e.)
Sleep start (hh:mm)	23:32 ± 00:06	23:42 ± 00:07	23:38 ± 00:03
Sleep end (hh:mm)	06:16 ± 00:05^a,c^	07:01 ± 00:05^b, c^	07:27 ± 00:05^a,b^
Time in bed (hh:mm)	06:59 ± 00:06^d,e^	07:37 ± 00:07^d^	08:08 ± 00:05^e^
Assumed sleep (hh:mm)	06:50 ± 00:06^f,g^	07:26 ± 00:07^f^	07:55 ± 00:05^g^
Actual sleep time (hh:mm)	05:27 ± 00:06^h^	05:59 ± 00:07	06:18 ± 00:06^h^
Actual sleep (%)	81.11 ± 0.78	82.11 ± 0.81	80.83 ± 0.76
Actual wake time (hh:mm)	01:15 ± 00:03	01:17 ± 00:03	01:28 ± 00:03
Actual wake (%)	18.89 ± 0.91	17.89 ± 0.81	18.17 ± 0.76
Sleep efficiency (%)	78.1 ± 1.06	78.91 ± 1.1	77.64 ± 0.92
Sleep latency (hh:mm)	00:08 ± 00:01	00:11 ± 00:01	00:12 ± 00:02
Immobile minutes (n)	396.32 ± 7.29^i,j^	390.9 ± 7.84	417.54 ± 6.77^i,j^
Immobile time (%)	82.02 ± 1.59	86.02 ± 1.15	83.86 ± 1.22
Mobile minutes (n)	37.77 ± 2.74	38.71 ± 2.18	45.5 ± 2.05
Mobile time (%)	10.29 ± 0.72	9.32 ± 0.51	10.38 ± 0.44
Fragmentation index (a.u.)	32.54 ± 1.99	30.93 ± 1.62	34.23 ± 1.74

a.u., arbitrary unit. ^a,e,g,h,i^Significant Bonferroni *post-hoc* test between very early and late mud application (*p* < 0.001; *p* < 0.001; *p* < 0.001; *p* < 0.001; *p* < 0.001, respectively). ^b^Significant Bonferroni *post-hoc* test between early and late mud application (*p* = 0.014). ^c,d,f^Significant Bonferroni *post-hoc* test between very early and early mud application (*p* < 0.001; *p* = 0.017; *p* = 0.038; *p* = 0.014, respectively).

Mud application timing appeared to lead to earlier wake-up times, reducing time spent in bed. However, the reduced sleep duration did not negatively affect sleep quality parameters. Thus, participants who had an early mud application slept less, but not worse, than those who had it later in the morning. Given these findings, the subsequent analysis assessed the daytime physical activity and daily duration of thermal-water bathing as potential main effects of sleep quality, while mud application time was considered a covariate.

### 3.4 Effect of daytime physical activity on the delta values of sleep parameters

**Figure 1:**
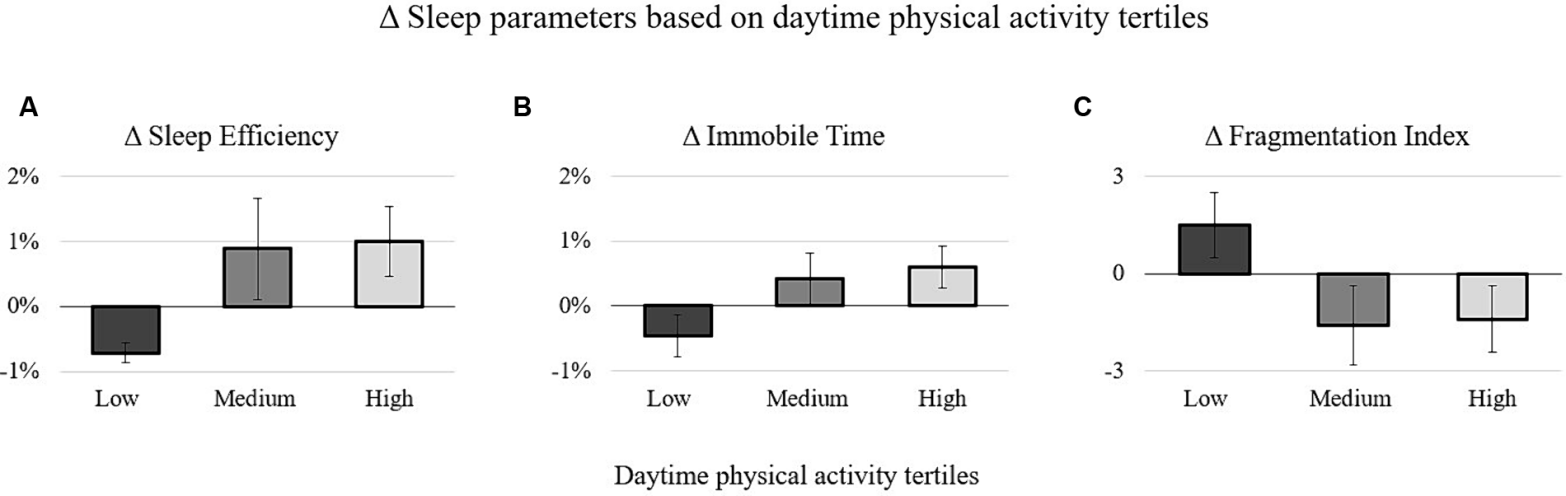
Delta values of the sleep efficiency **(A)**, immobile time **(B)**, and fragmentation index **(C)** stratified for the tertiles of the daytime physical activity.

The two-way ANCOVA revealed a significant effect of daytime physical activity on Δ *Sleep Efficiency* [*F*_(2, 125)_ = 2.8, *p* < 0.05, *η_p_^2^* = 0.05]. Although no *post-hoc* tests yielded statistically significant differences for the effect of daytime physical activity on Δ *Sleep Efficiency*, it is worth noting that individuals with *high* daytime physical activity exhibited a 1% increase in Δ *Sleep Efficiency* compared to those with *low* daytime physical activity, who, conversely, experienced a decrease in *Sleep Efficiency* (Table 2; Figure 1A). Despite the lack of statistical significance, individuals with *medium* and *high* daytime physical activity tended to show slight improvements in sleep parameters compared to those with *low* daytime physical activity (Table 2; Figures 1B,C). Therefore, it seems that daytime physical activity could have an influence on sleep efficiency with a tendency to improve it. In fact, even though it was not significant, participants who were more active during the day tended to improve their sleep quality and sleep slightly better than those with less active daily routines.

**Table 2:** Mean and standard error of the sleep parameters stratified for the tertiles of the daytime physical activity and daily duration of thermal water bathing.

Δ Sleep parameters	Tertiles					
	Daytime physical activity			Daily duration of thermal-water bathing		
	Low (Mean ± s.e.)	Medium (Mean ± s.e.)	High (Mean ± s.e.)	Short (Mean ± s.e.)	Intermediate (Mean ± s.e.)	Long (Mean ± s.e.)
Sleep start (hh:mm)	00:14 ± 00:04	00:17 ± 00:04	00:12 ± 00:05	00:11 ± 00:04	00:16 ± 00:04	00:15 ± 00:04
Sleep end (hh:mm)	00:03 ± 00:03	−00:03 ± 00:02	00:03 ± 00:03	−00:05 ± 00:03	00:04 ± 00:03	00:04 ± 00:03
Time in bed (hh:mm)	−00:11 ± 00:04	−00:23 ± 00:05	−00:13 ± 00:05	−00:18 ± 00:05	−00:12 ± 00:05	−00:17 ± 00:05
Assumed sleep (hh:mm)	−00:10 ± 00:06	−00:16 ± 00:06	−00:08 ± 00:06	−00:16 ± 00:05	−00:11 ± 00:06	−00:15 ± 00:05
Actual sleep time (hh:mm)	−00:09 ± 00:04	−00:13 ± 00:05	−00:05 ± 00:04	−00:15 ± 00:12	−00:09 ± 00:08	−00:02 ± 00:02
Actual sleep (%)	−0.64 ± 0.11	−0.57 ± 0.17	0.84 ± 0.53	−0.6 ± 0.57^a^	−0.14 ± 0.11	1.5 ± 0.6^a^
Actual wake time (hh:mm)	−00:03 ± 00:01	−00:04 ± 00:02	−00:06 ± 00:02	−00:03 ± 00:02	−00:02 ± 00:02	−00:08 ± 00:02
Actual wake (%)	0.64 ± 0.11	0.57 ± 0.17	−0.84 ± 0.53	0.6 ± 0.57^b^	0.14 ± 0.6	−1.5 ± 0.6^b^
Sleep efficiency (%)	−0.71 ± 0.15	0.89 ± 0.78	1 ± 0.53	−0.34 ± 0.31^c^	−0.11 ± 0.09	2.15 ± 0.9^c^
Sleep latency (hh:mm)	00:02 ± 00:01	−00:02 ± 00:01	−00:01 ± 00:01	−00:01 ± 00:00	−00:03 ± 00:01	−00:02 ± 00:01
Immobile minutes (n)	−11.34 ± 4.77	−14.62 ± 5.18	−8 ± 5.6	−15.25 ± 5.07	−11.38 ± 5.35	−5.8 ± 4.81
Immobile time (%)	−0.46 ± 0.33	0.42 ± 0.40	0.6 ± 0.32	−0.21 ± 0.19^d^	−0.15 ± 0.1	1.15 ± 0.43^d^
Mobile minutes (n)	2.19 ± 0.17	−3.39 ± 1.7	−4.07 ± 1.55	−0.39 ± 1.71	−0.83 ± 1.87	−6.32 ± 2.11
Mobile time (%)	0.46 ± 0.33	−0.42 ± 0.40	−0.6 ± 0.32	0.21 ± 0.37^e^	0.15 ± 0.44	−1.15 ± 0.43^e^
Fragmentation index (a.u.)	1.5 ± 1	−1.6 ± 1.21	−1.4 ± 1.03	0.88 ± 0.75	0.55 ± 0.43	−2.98 ± 1.34

a.u., arbitrary unit. ^a,b,c,d,e^Significant Bonferroni *post-hoc* test between short and long daily thermal-water bathing (*p* = 0.04, *p* = 0.04, *p* < 0.007, *p* = 0.05, *p* = 0.05, respectively).

### 3.5 Effect of thermal-water-bathing duration on the delta values of sleep parameters

The two-way ANCOVA revealed significant effect of daily duration of the thermal-water-bathing on the values of Δ *Actual sleep time* (%) [*F*_(2, 125)_ = 3.22, *p* < 0.044, *ƞ_p_*^2^ = 0.06], Δ *Actual wake time* (%) [*F*_(2, 125)_ = 3.21, *p* < 0.044, *ƞ_p_*^2^ = 0.06], Δ *Sleep efficiency* [*F*_(2, 125)_ = 5.01, *p* < 0.008, *ƞ_p_*^2^ = 0.09], Δ *Immobile time* (%) [*F*_(2, 125)_ = 3.31, *p* < 0.04, *ƞ_p_*^2^ = 0.06], and Δ *Mobile time* (%) [*F*_(2, 125)_ = 3.31, *p* < 0.04, *ƞ_p_*^2^ = 0.06].

**Figure 2:**
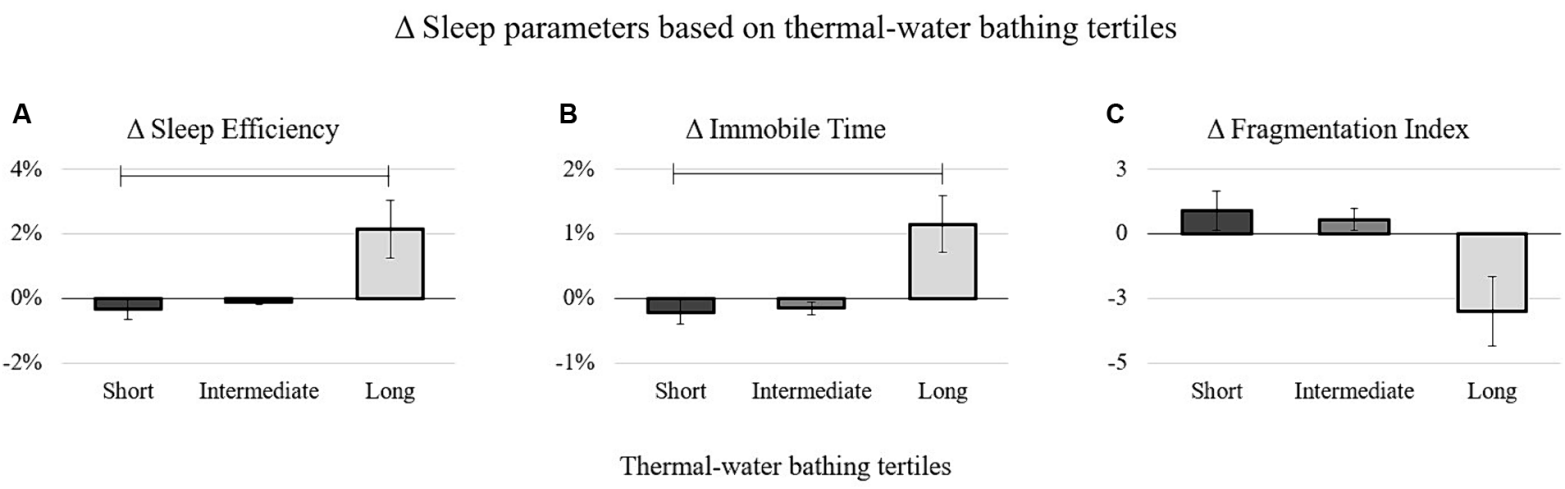
Delta values of the sleep efficiency **(A)**, Immobile time **(B)**, and fragmentation index **(C)** stratified for the tertiles of the daily duration of thermal-water bathing. The bars in **(A,B)** indicate statistically significant differences between *long* and *short* daily bathing duration in thermal-water pools.

More specifically, the Bonferroni *post-hoc* tests revealed that participants with *long* daily thermal-water bathing duration increased the percentage of time spent sleeping (*p* = 0.04*, d =* 0.61), sleep efficiency (*p* = 0.007*, d =* 0.5) (Figure 2A), and the percentage of time in immobility while sleeping (*p* = 0.05*, d =* 0.47) (Figure 2B), whereas they reduced the percentage of awake periods (*p* = 0.04*, d =* −0.61) and mobile periods while sleeping (*p* = 0.05*, d =* 0.47) compared to those having *short* daily thermal-water bathing duration. Furthermore, participants with *long* daily thermal-water bathing duration tended to improve sleep efficiency more than those with *intermediate* daily thermal-water bathing duration (*p* = 0.07, *d* = 0.43) (Table 2).

Additionally, a trend toward significance was observed for the effect of daily thermal-water bathing duration on Δ *Mobile Minutes (%)* [*F*_(2, 125)_ = 2.79, *p* < 0.06, *η_p_^2^* = 0.05] and Δ *Fragmentation Index* [*F*_(2, 125)_ = 2.5, *p* < 0.07, *η_p_^2^* = 0.04]. Although not reaching statistical significance, participants with *long* daily thermal-water bathing duration displayed the highest decrease in the mobile minutes while sleeping and the level of sleep fragmentation (Table 2; Figure 2C).

In summary, independently of the wake-up time due to mud application, participants who had longer daily baths in thermal-water pools improved their sleep quality more than those who bathed shortly during the day.

## 4 Discussion

The current study investigates changes in sleep quality among 127 healthy Italian subjects during a 1 week of spa stay and balneotherapy in Abano Terme, Italy. Sleep quality was examined considering the influence of daytime physical activity and balneotherapy, including mud applications and bathing in thermal-water pools. In the current study, results indicate that sleep characteristics were affected differently by daytime physical activity, thermal-water bathing, and mud applications. Daytime physical activity seems to have minimal impact on sleep, whereas thermal-water bathing was associated with improved sleep quality, particularly when it lasted longer than 75 min. Conversely, mud applications primarily influenced sleep timings and duration, with participants having earlier appointments experiencing shorter sleep durations compared to others.

To the best of our knowledge, this is the first study assessing the effects of daytime physical activity and balneotherapy on sleep quality in a sample of healthy subjects during a week of spa stay. The effect of daytime physical activity arising from our data is not clear-cut. Precedent studies that objectively evaluated daytime physical activity and sleep highlighted that subjects with a more active daily routine also reported a longer total sleep time (24, 25), fewer awakenings after sleep onset (31), and a lower risk of reduced sleep quality (26, 27). We are not able to draw the same conclusions from our results, even though participants who had a more active daily routine increased their sleep efficiency by 1%.

Regarding balneotherapy, previous studies focused on monitoring sleep quality changes in unhealthy subjects undergoing balneotherapy treatments. In patients with musculoskeletal pathologies or psychological disorders, improved sleep following balneotherapy treatments could be attributed to relief in pathology symptoms, which may disturb sleep less (32–42). However, in healthy individuals, the previous explanation is unreliable to account for sleep improvements. Therefore, we propose some hypotheses below to elucidate our sleep results in sections 4.2 and 4.3.

### 4.1 Daytime physical activity effect on sleep

Even though we missed an apparent effect of higher daytime physical activity connected to better sleep quality, the current data may suggest that being more active during the day could offer the possibility to sleep slightly better compared to subjects with a less active daily routine. Since highlighting a connection between daytime physical activity and sleep was one of our aims, we later explained some possible explanations for this connection, which could be the basis for future studies aiming at pointing out if and how daytime physical activity could play a role in influencing sleep quality.

A positive feedback loop between daytime physical activity and sleep quality could be hypothesized. More active individuals may experience better sleep, leading to increased energy levels and a greater inclination for physical activity the following day. Conversely, less active individuals may not see improvements in their sleep quality, potentially creating a cycle of lower physical activity and unchanged sleep quality (49, 50).

Several hypotheses can explain potential connections between daytime physical activity and sleep. Firstly, individuals who engage in higher activity levels during the day may experience a greater need for recovery due to increased energy expenditure (49, 50). Secondly, improved thermoregulation may occur, as heightened daytime physical activity could elevate body temperature, leading to a more significant drop during the night and facilitating sleep onset (49). Lastly, an active daily routine might contribute to lower anxiety and stress levels, potentially resulting in less disturbed sleep and, consequently, better sleep quality (51).

The sleep window during the 24 h is intrinsically correlated to the rest-activity circadian rhythm. Epidemiological studies in older adults have not only demonstrated the positive influence of structured physical activity but also suggested that daytime (non-structured) physical activity could regulate the rest-activity circadian rhythm, positively impacting sleep quality (29, 52). Additionally, a more active daytime routine is believed to enhance physical functioning, which has recently been identified as a mediator between daytime physical activity and sleep quality (27).

### 4.2 Mud application effect on sleep

The effects of balneotherapy on sleep quality vary depending on the specific thermal treatments considered. In our study, the impact of mud applications was assessed solely based on their administration time, as all patients underwent the same mud application protocol, and there was no control group without mud application.

The timing of mud application influenced both sleep timing and duration. Early mud application, particularly before 8:30 a.m., led to earlier wake-up times compared to later treatments, with a more pronounced effect for those with mud applications fixed before 7:30 a.m. Regarding sleep duration, the anticipated wake-up time reduced the time spent in bed and, consequently, the minutes of sleep and immobility.

However, no differences in sleep duration parameters expressed in percentages or indicators of sleep quality were observed based on the timing of mud application. Therefore, those with earlier mud applications experienced a reduction in sleep duration without a corresponding decrease in sleep quality, underscoring that longer sleep duration does not necessarily equate to better sleep quality (53, 54).

Nevertheless, the presumption that mud application in the late morning has no impact on sleep quality has not been foregone. Despite the unclear effect of mud application on cortisol secretion (55), studies by Tanizaki et al., Ortega et al., and the recent review by Gàlvez et al. have reported an increase in cortisol levels following mud application (56–58). Elevated cortisol secretion or its misalignment with normal circadian rhythms could potentially disrupt the rest-activity circadian rhythm and negatively affect sleep quality (59, 60). However, in our current study, whether mud application occurred early (at 6:00 a.m.) or late (at 11:20 a.m.), there seems to be no discernible positive or negative effect on sleep quality.

### 4.3 Duration of bathing in thermal-water pool effect on sleep

Moving on to the second thermal treatment assessed—bathing duration in thermal-water pools—analyses suggest a potential association with more significant improvements in sleep quality. Participants who bathed for longer than 75 min per day showed a more marked enhancement in sleep quality than those bathing for less than 55 min. The former reported more significant improvements in sleep efficiency and time spent in immobility and mobility while sleeping, resulting in a more substantial amelioration of sleep fragmentation.

To the best of our knowledge, this study is the first to objectively assess the impact of bathing duration in thermal-water pools on sleep quality in healthy Italian subjects. Previous studies focused on patients with various pathologies, experiencing stress or burnout, where improvements in sleep were primarily associated with pain or symptom relief (34–36). Additionally, these studies often relied on questionnaires or subjective evaluations to assess sleep quality, while our study employed actigraphy for objective sleep assessment. Our findings suggest that daily thermal-water bathing for more than 75 min could potentially contribute to enhanced objectively assessed sleep quality. In the quest for non-invasive and non-pharmacological solutions to improve sleep, thermal-water bathing emerges as a promising alternative.

While we did not investigate the mechanical or physiological pathways that account for the impact of thermal-water bathing on sleep quality, we can propose some potential hypotheses. Two of these hypotheses share similarities with those suggested to explain the effects of daytime physical activity and are based on water’s thermal, mechanical, and chemical pathways (61).

Firstly, bathing in thermal-water pools might have exerted a relaxing and anti-stress effect, positively influencing sleep. The calming properties of thermal water have been extensively documented in the literature (62, 63), at times linked to a reduction in cortisol secretion (55), and more recently observed in a cohort of healthy adults (64). The reduction of anxiety and stress levels is crucial for enhancing sleep quality (5).

Secondly, consistent and daily immersion in thermal water at temperatures of 32–36°C could have affected body temperature through a cycle of increase followed by decrease. This body temperature fluctuation, primarily induced by peripheral vasodilation (65–67), could have facilitated the initiation and maintenance of sleep. Several studies in diverse populations have put forth this hypothesis to explain changes in sleep patterns (40, 68).

Thirdly, the vasodilation induced by bathing in 32–36-degree thermal water could also contribute to improved systemic blood circulation and cardiovascular function. The combination of heat and the hydrostatic pressure of the water may enhance cardiovascular function by redirecting blood flow to the main blood vessels (65). Impaired cardiovascular function is often linked to sleep disorders, particularly difficulties in initiating sleep and insomnia, and these factors are intricately connected bidirectionally (69, 70).

These results could raise awareness among medical professionals working in spa or thermal centers about the potential of balneotherapy in relieving sleep problems. Hydrologist medical doctors may suggest best practices to ensure their clients benefit from balneotherapy treatments. Additionally, general doctors and practitioners could consider these results when advising their patients about the potential benefits of a spa stay and an active daily routine to improve sleep quality.

The findings of this study can be understood in the context of its strengths and limitations. Noteworthy strengths include (i) the unique characteristics of the population, focusing on a healthy group—an aspect seldom explored in this field, (ii) the objective measurement of daytime physical activity and sleep parameters, a feature frequently absent in previous research, and (iii) the novelty of results indicating the potential positive impact of thermal-water bathing on sleep quality in healthy individuals. However, there are limitations, including (i) the absence of a control group and/or unhealthy group (though it is almost impossible to recruit customers not undergoing spa treatments during a spa holiday), (ii) the small sample size and its old age, which limit the generalizability to younger subjects (iii) the recruitment of only Italian participants, and (iv) the lack of assessment of daytime physical activity before and after the weekly spa stay. Moreover, some biases and confounding factors could be present, such as different seasons of the year with varying light exposure during recruitment, the single recruitment place with a specific mud application protocol and times, which could differ from other spa resorts, and the absence of monitoring of electronic device and light exposure.

Future studies should aim to validate and expand upon these findings with larger sample sizes and seek more definitive explanations for the observed trend of thermal bathings improving the qualitative aspects of sleep. Future research could propose assessments of melatonin and cortisol levels, as well as body temperature, to find plausible explanations for changes in sleep quality. Another possible future area of study could be the comparison of different age groups to assess whether balneotherapy and daytime physical activity may have similar effects on sleep quality regardless of age.

## 5 Conclusion

Spending a week at a spa with thermal-water bathings longer than 75 min per day appears to have positive and beneficial effects on sleep, contributing to improving sleep quality during the stay. Early morning mud applications may shorten sleep duration without compromising sleep quality. Thus, thermal-water bathings emerge as a potentially effective balneotherapy treatment for enhancing sleep quality in healthy individuals. Along with thermal-water bathings, maintaining an active routine during the day may further improve sleep quality. The current findings can lay the foundations for recommending balneotherapy, typically prescribed for individuals with mild-to-severe musculoskeletal pathologies or stress-related disorders, even in healthy subjects seeking to enhance their sleep quality.

## Data availability statement

The original contributions presented in the study are included in the article/Supplementary material, further inquiries can be directed to the corresponding author.

## Ethics statement

The studies involving humans were approved by University of Milan’s Ethical Committee (24/20). The studies were conducted in accordance with the local legislation and institutional requirements. The participants provided their written informed consent to participate in this study.

## Author contributions

LC: Data curation, Formal analysis, Investigation, Methodology, Writing – original draft, Writing – review & editing. AC: Investigation, Writing – original draft, Writing – review & editing. LG: Formal analysis, Investigation, Methodology, Writing – original draft, Writing – review & editing. AMu: Investigation, Methodology, Writing – review & editing. FF: Investigation, Methodology, Supervision, Writing – review & editing. AMo: Conceptualization, Methodology, Project administration, Resources, Supervision, Writing – review & editing. ER: Conceptualization, Methodology, Project administration, Resources, Supervision, Writing – review & editing. FE: Conceptualization, Funding acquisition, Project administration, Resources, Supervision, Writing – review & editing.
