# Supplementary figures and images for: Revitalizing your sleep: the impact of daytime physical activity and balneotherapy during a spa stay
Source: Front Public Health. 2024 Jul 8;12:1339689. doi: 10.3389/fpubh.2024.1339689 (PMC11267379; doi:10.3389/fpubh.2024.1339689)

## FLOW CHART OF THE STUDY PROTOCOL

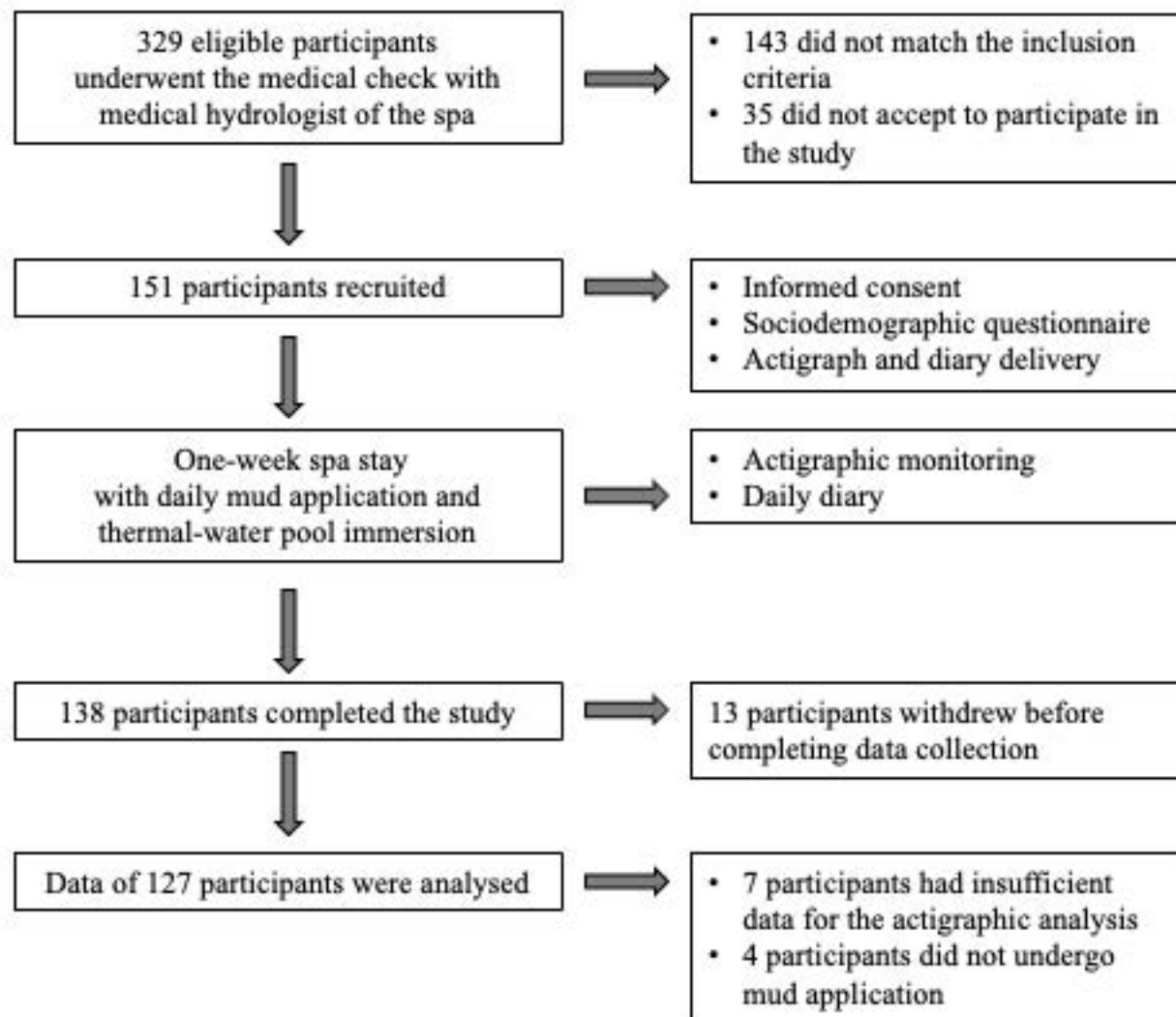

Supplement: Supplementary file 1 [file Data_Sheet_1.pdf]
