# Supplementary material for: Revitalizing your sleep: the impact of daytime physical activity and balneotherapy during a spa stay
Source: Front Public Health. 2024 Jul 8;12:1339689. doi: 10.3389/fpubh.2024.1339689 (PMC11267379; doi:10.3389/fpubh.2024.1339689)
Supplement: Supplementary file 2 [file Data_Sheet_2.docx]

- 1. *Sleep parameters assessment*

The evaluated sleep parameters are ^1^:

- *Sleep Start*: marks the start of sleep based on detected movements below a set threshold;
- *Sleep End*: marks the end of the sleep based on detected movements above a set threshold;
- *Time in Bed*: the time, expressed in hours and minutes, spent in bed between bedtime and wake-up time; therefore, includes time spent sleeping and awake (e.g. sleep latency);
- *Assumed Sleep*: the actual time, expressed in hours and minutes, between the beginning and the end of the sleep; therefore, does not include sleep latency;
- *Actual Sleep Time*: the time, expressed in hours and minutes, spent in sleep, thus removing from Assumed Sleep the time spent in wakefulness;
- *Actual Sleep Time (%)*: the sleep time expressed as a percentage of Assumed Sleep;
- *Actual Wake Time*: is the time, expressed in hours and minutes, spent in wakefulness;
- *Actual Wake Time (%)*: the time spent in wakefulness expressed as a percentage of Assumed Sleep;
- *Sleep Efficiency (%)*: the Actual Sleep Time expressed as a percentage of the Time in Bed;
- *Sleep Latency*: the time, expressed in hours and minutes, between bedtime and Sleep start;
- *Immobile Minutes*: the number of minutes spent in immobility during Assumed Sleep;
- *Immobile Time (%)*: the number of immobile minutes expressed as a percentage of Assumed Sleep;
- *Moving Minutes*: the number of minutes with detected movements during Assumed Sleep;
- *Moving Time(%)*: the number of minutes of movement expressed as a percentage of Assumed Sleep;
- *Fragmentation Index:* is an index that describes sleep fragmentation.
  1. *Mud application*

The north-eastern Italian Euganean Thermal District, which includes the thermal locality of Abano Terme, is internationally renowned for the composition and quality of its natural mud. After being extracted from two Euganean lakes, mud undergoes a tank-mature system lasting between two and six months. Each spa is responsible for complying with the disciplinary for the correct natural maturation of the mud, avoiding using bentonite and other clays. At the end of the tank-mature system, muds are rich in cyanobacteria and diatoms, which the Italian Health System recognises to be relieving for rheumatic diseases ^2–4^.

Mud application follows different protocols depending on the pathology of the patient; since our participants did not show rheumatic or other pathologies but wanted to have mud applications for preventive reasons, the doctor prescribed a fifteen-minute-mud-application protocol followed by a ten-minute-thermal-water hydromassage.

*2.3 Bathing in thermal-water pools*

Natural thermal springs in the Euganean Thermal District have a source temperature of around 75° C and total dissolved solids between 2.3 and 6 g/L (principal minerals: chlorine, sodium, potassium, magnesium, sulphur, bromide, iodine and silicon dioxide). Thus, Euganean thermal water is a hyperthermic, hypersaline, and salty-bromine-iodine thermal water. The water temperature is cooled to 32-36°C in the thermal-water pools ^2,4^.

Bathing in the thermal-water pools was free: each participant decided when and how long to bathe, swim or do acquagym courses during the day. Some participants received prescriptions for personalised hydrokinesis therapy in thermal water from the medical doctor. We collected daily thermal-water pools' time and duration from the daily diary.

1. Galasso L, Calogiuri G, Castelli L, Mulè A, Esposito F, Caumo A, et al. Theoretical construct into blocks of actigraphic-derived sleep parameters. Chronobiol Int [Internet]. 2023 Feb 1;40(2):174–85. Available from: https://doi.org/10.1080/07420528.2022.2157737

2. Gris B, Treu L, Zampieri RM, Caldara F, Romualdi C, Campanaro S, et al. Microbiota of the therapeutic Euganean thermal muds with a focus on the main cyanobacteria species. Microorganisms. 2020 Oct 1;8(10):1–23.

3. Baldovin T, Amoruso I, Caldara F, Buja A, Baldo V, Cocchio S, et al. Microbiological hygiene quality of thermal muds: A pilot study in pelotherapy facilities of the euganean thermal district (ne italy). Int J Environ Res Public Health. 2020 Jul 2;17(14):1–15.

4. Camera del Commercio di Padova. Marchio collettivo “Fango D.O.C. – Thermae Abano Montegrotto – Regione Veneto.” https://www.pd.camcom.it/it/tutela-impresa-e-consumatore/marchi-e-brevetti-1/marchio-collettivo-fango. 2022.
