# Supplementary material for: Revitalizing your sleep: the impact of daytime physical activity and balneotherapy during a spa stay
Source: Front Public Health. 2024 Jul 8;12:1339689. doi: 10.3389/fpubh.2024.1339689 (PMC11267379; doi:10.3389/fpubh.2024.1339689)
Supplement: Supplementary file 3 [file Data_Sheet_3.docx]

**Table 1** Descriptive data in the total sample (N = 127) reported as mean ± SD, number and percentage.

| **Variable** | **Mean ± s.e.** | | **N (%)** | |
| --- | --- | --- | --- | --- |
| Age (years) | 64.61 ± 0.89 |  | |  |
| Body mass (kg) | 71.3 ± 1.31 |  | |  |
| Statura (m) | 1.68 ± 0.01 |  | |  |
| BMI (kg/m^2^) | 25.21 ± 0.39 |  | |  |
| Normal weight |  | 65 (51.1%) | |  |
| Overweight |  | 43 (33.9%) | |  |
| Obese |  | 19 (15%) | |  |
| Sex |  |  | |  |
| Male |  | 44 (34.6%) | |  |
| Female |  | 83 (65.4%) | |  |
| Education level |  |  | |  |
| Primary education diploma |  | 8 (6.3) | |  |
| Secondary education diploma |  | 64 (50.4) | |  |
| Bachelor's degree |  | 7 (5.5) | |  |
| Master's degree |  | 35 (27.6) | |  |
| Master or specialisation |  | 7 (5.5) | |  |
| Ph.D. |  | 2 (1.6) | |  |
| Other |  | 4 (3.1) | |  |
| Marital status |  |  | |  |
| Single |  | 16 (12.6%) | |  |
| Married |  | 72 (56.7%) | |  |
| Cohabitant |  | 11 (8.7%) | |  |
| Divorced |  | 16 (12.6%) | |  |
| Widow/Widower |  | 11 (8.7%) | |  |

**Table 2** Mean weekly values of the daytime activity, sleep parameters, and balneotherapy

| **Variable** | **Mean ± s.e.** |
| --- | --- |
| Daytime physical activity (%) | 42.45 ± 0.75 |
| Sleep Start (hh:mm) | 23:36 ± 00:03 |
| Sleep End (hh:mm) | 06:54 ± 00:03 |
| Time In Bed (hh:mm) | 07:34 ± 00:04 |
| Assumed Sleep (hh:mm) | 07:23 ± 00:04 |
| Actual Sleep Time (hh:mm) | 05:54 ± 00:04 |
| Actual Sleep (%) | 81.37 ± 0.48 |
| Actual Wake Time (hh:mm) | 01:20 ± 0:02 |
| Actual Wake (%) | 18.63 ± 0.48 |
| Sleep Efficiency (%) | 78.24 ± 0.58 |
| Sleep Latency (hh:mm) | 00:10 ± 00:01 |
| Immobile Minutes (n) | 388.39 ± 4.58 |
| Immobile Time (%) | 83.99 ± 0.71 |
| Mobile Minutes (n) | 40.55 ± 1.37 |
| Mobile Time (%) | 9.98 ± 0.32 |
| Fragmentation Index (a.u.) | 32.52 ± 0.99 |
| Time of mud application (hh:mm) | 8:21 ± 0:10 |
| Daily duration of thermal-water bathing (minutes) | 68.44 ± 2.7 |

a.u. = arbitrary unit.
